# Supplementary material for: Glial granules contain germline proteins in the Drosophila brain, which regulate brain transcriptome
Source: Commun Biol. 2020 Nov 20;3:699. doi: 10.1038/s42003-020-01432-z (PMC7679405; doi:10.1038/s42003-020-01432-z)
Supplement: Supplementary file 1 — Supplementary Information [file 42003_2020_1432_MOESM1_ESM.pdf]

Supplementary Figures

Glial granules contain germline proteins in the *Drosophila* brain, which regulate brain transcriptome

Samuel J. Tindell[1], Eric C. Rouchka[2] & Alexey L. Arkov[1*]

[1]Department of Biological Sciences, Murray State University, Murray, KY, 42071, USA
[2]Computer Science and Engineering Department, University of Louisville, Louisville, KY, 40292, USA

[*]email: aarkov@murraystate.edu

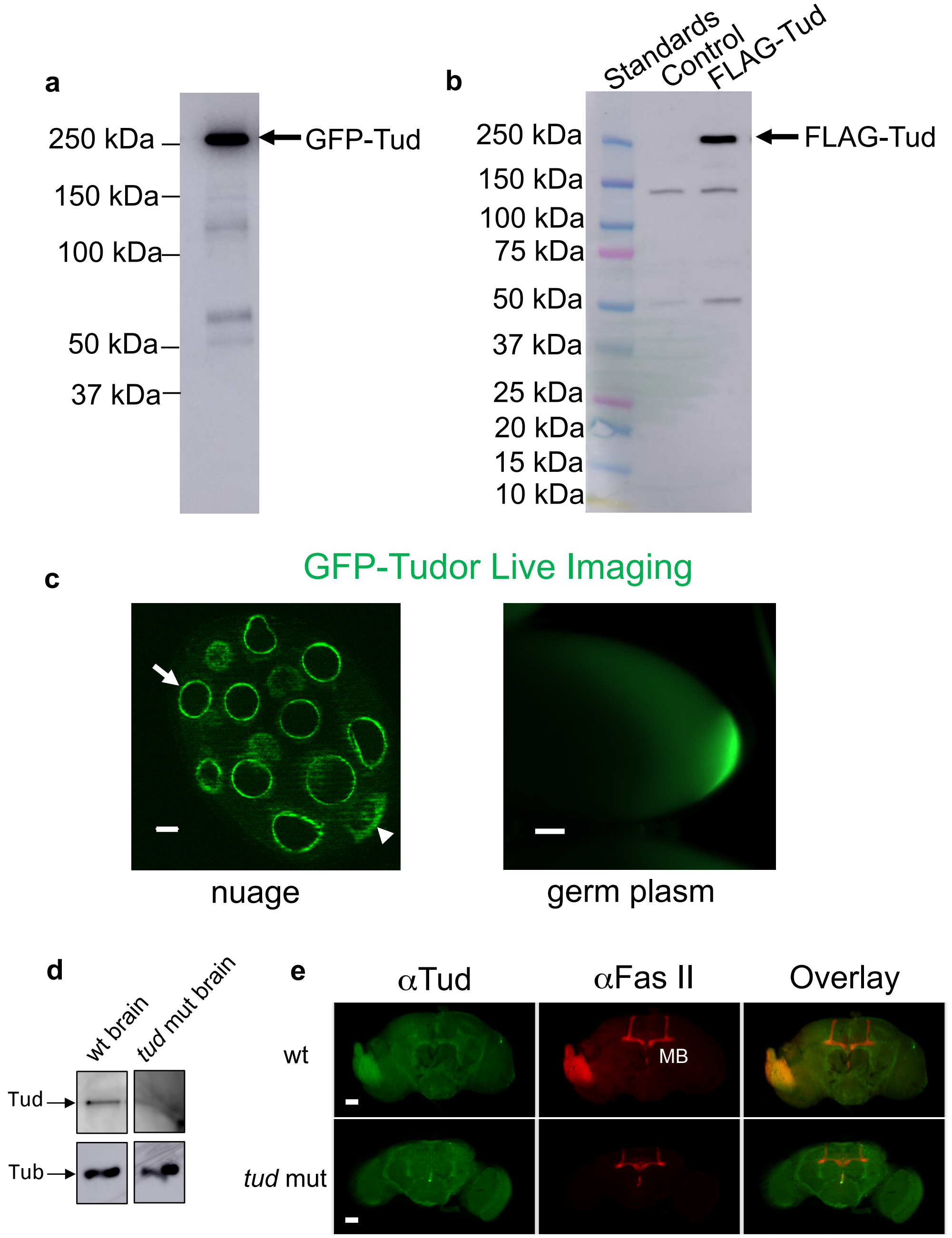

Supplementary Fig. 1: Endogenous GFP- and FLAG-tagged Tudor show characteristic expression in *Drosophila*. **a**, **b** Western blots showing expression of GFP- and FLAG-tagged endogenous Tud generated with CRISPR-Cas9 methodology in the ovary detected with anti-GFP and anti-FLAG antibodies (**a** and **b** respectively). In **b**, extract from non-tagged wild-type ovaries was used as a negative control. **c** Live imaging shows GFP-tagged endogenous Tud characteristic localization to perinuclear nuage in nurse cells (arrow) in the egg chamber (left panel). Also, the accumulation of Tud in the early-stage oocyte at the egg chamber's posterior is seen (arrowhead). Subsequent localization of the GFP-Tud to the late-stage oocyte's posterior germ plasm is shown on the right panel. **d** Western blot showing Tud expression in *Drosophila* brain with anti-

Tud antibody. Tud was immunoprecipitated from the wild-type (wt) fly brain lysates with anti-Tud antibody and detected using western-blot technique with the same antibody. As expected, no Tud was detected in the brain lysates from *tud* null (*tud*^{1}) flies (*tud* mut); top images from the same gel are shown. The same total protein concentration in both wt and *tud* null brain lysates was used in these experiments as confirmed by western-blot procedure using anti-β-Tubulin (Tub) antibody. **e** Brain immunostaining with anti-Tud antibody does not provide adequate signal-to-noise ratio to detect Tud expression. Staining of wild-type (wt) and *tud* null mutant brains with anti-Tud (green channel) and anti-Fasciclin II (Fas II, red channel) antibodies. Anti-Fas II antibody labels the mushroom body (MB) neurons in both wt and *tud* mutant brains. Scale bars in left and right panels in **c** are 10 μm and 30 μm respectively, and in **e** 50 μm.

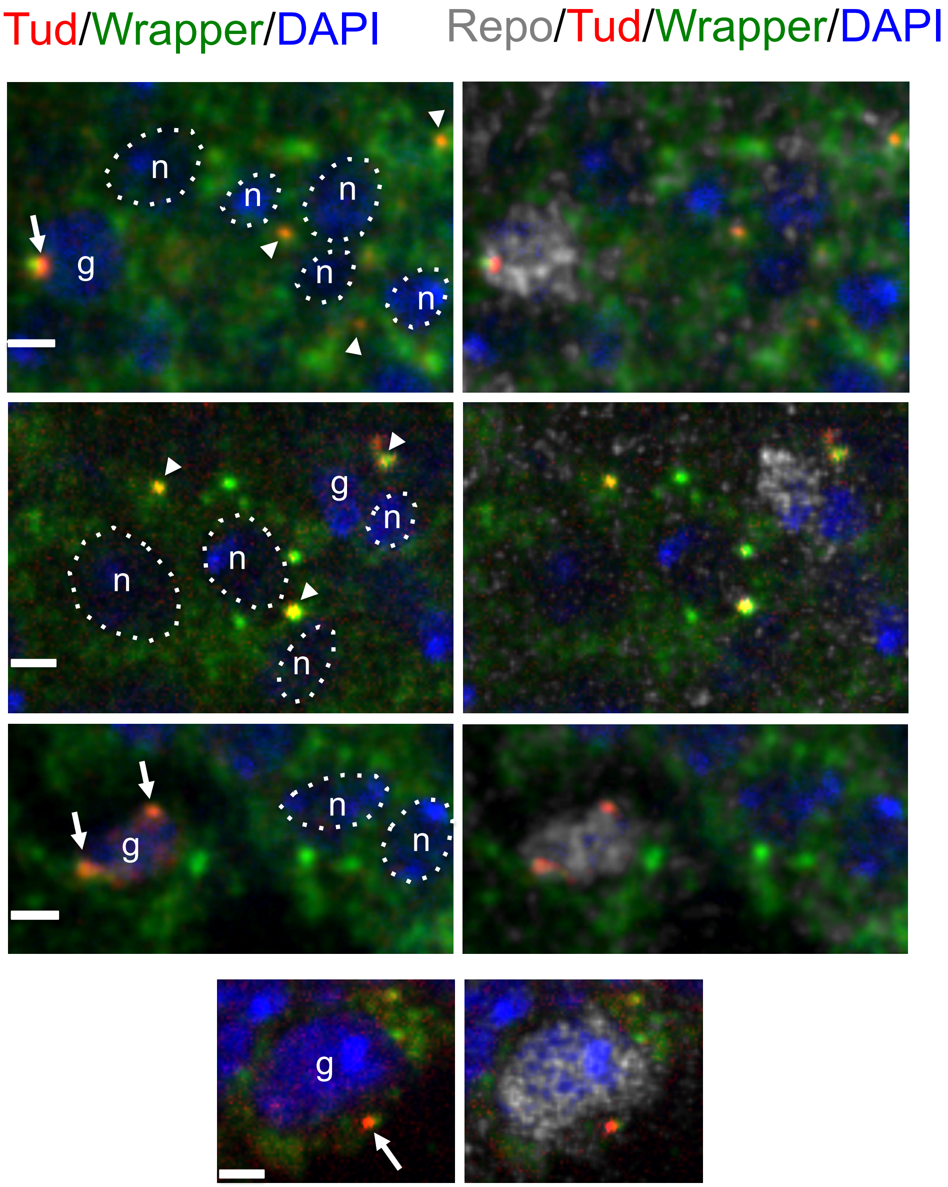

Supplementary Fig. 2: Details of Tudor expression in cortex glia. Optical sections show cortex glial cells labeled with anti-Wrapper antibody (cortex glial cell membrane

and cortex glial processes, green channel) and anti-Repo antibody (glial cell's nuclei, gray). DAPI stains nuclei and Repo-negative/DAPI-positive nuclei are those of neuronal bodies tightly surrounded by cortex glial processes labeled with anti-Wrapper. Tudor granules are labeled with anti-FLAG antibody (red channel). In different rows, the images are different optical sections from central brain cortex indicated in Fig. 1b and for a section presented in each row, right panel includes Repo signal in addition to that of Tud/Wrapper/DAPI shown in the left panel. Glial nuclei are indicated with "g" and neuronal bodies are indicated with dotted line and "n". Tud granules are located either within the cortex glia processes (arrowheads) or close to cortex glia nucleus (arrows). Scale bars are 2 μm.

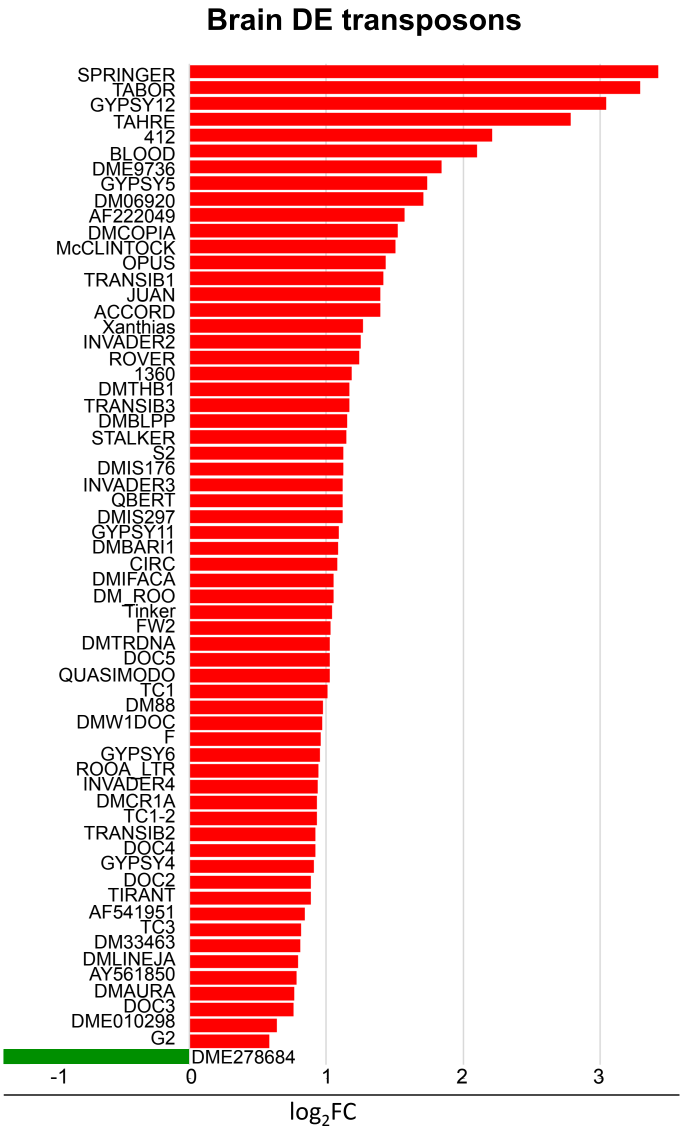

Supplementary Fig. 3: All transposable elements that are differentially expressed in *tud* mutant brains. 62 transposable elements are upregulated (red bars) and one is downregulated (green bar) in *tud* mutant brains based on a p-value of 0.05 (a subset of the most highly differentially expressed transposable elements is shown in Fig. 2a). X-

axis shows $\log_2$ values of changes in the transposon levels in *tud* mutants versus wild-type control. FC, fold change (source data are included in Supplementary Data 3).

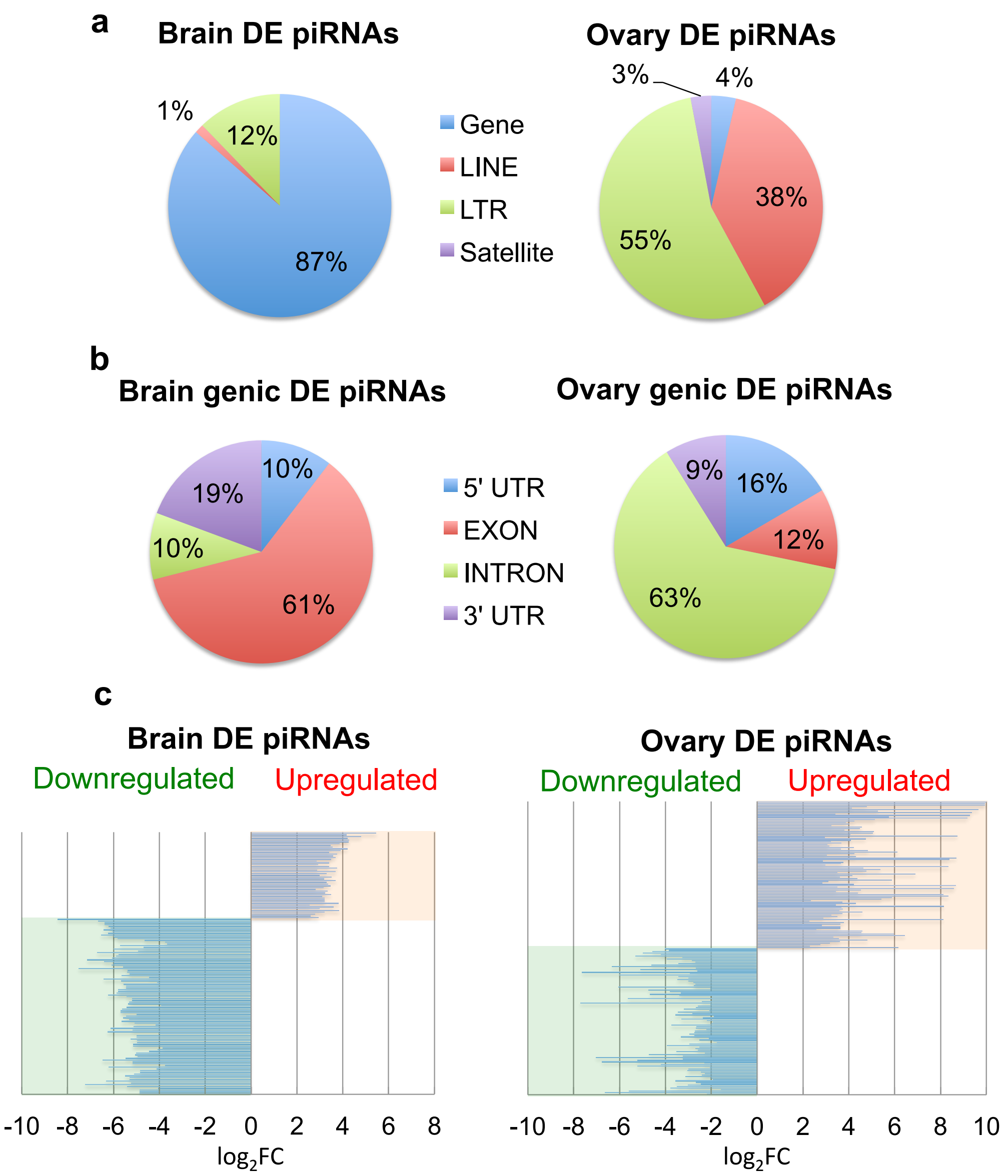

Supplementary Fig. 4: Contrary to ovarian piRNAs, piRNAs differentially expressed in *tud* mutant brains are downregulated to a higher degree than the upregulated piRNAs, and are mainly mapping to exons and 3′UTRs. a Pie charts show percentage distribution of piRNAs differentially expressed (DE) in *tud* mutant brains (left panel) and ovaries (right panel) mapped to non-transposon genes (genic piRNAs) or LTR and LINE transposons. In addition, a small fraction of ovarian DE piRNAs was mapped to satellite DNA. **b** Pie charts show the distribution of genic DE piRNAs within 5′ UTRs, exons, introns and 3′ UTRs in brains (left) and ovaries (right). **c** Most of the piRNAs differentially expressed in *tud* mutant brains are downregulated to a higher degree compared to upregulated piRNAs (left panel). This strong effect of *tud* mutation on the piRNA production in the brain is in contrast to a generally stronger effect

of *tud* mutation on the piRNA upregulation compared to the downregulation of piRNAs in the ovary (right panel). Log_2FC values (fold change, FC) for all 49 upregulated piRNAs and top 100 downregulated piRNAs in *tud* mutant brains and for top 100 upregulated and downregulated piRNAs in *tud* mutant ovaries are plotted. DE piRNA names, sequences, Log_2FC values and exact p- and FDR-values for each piRNA are provided in Supplementary Data 1 and 2.

a

Classification of DE genes in *tud* mutant brains

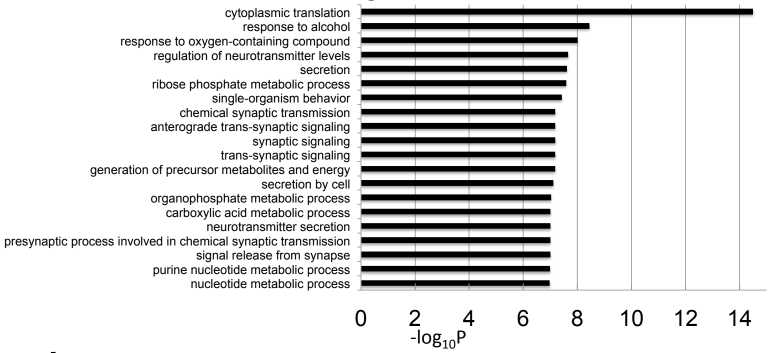

b

Classification of DE genes in *tud* mutant ovaries

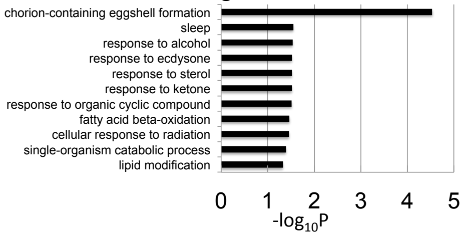

c

Enriched GO BP terms for DE genes in *tud* mutant brains

GO:BP ID	Description	P-value
GO:0002181	cytoplasmic translation	3.23970267475389e-15
GO:0097305	response to alcohol	3.58609255574044e-09
GO:1901700	response to oxygen-containing compound	9.82101915689245e-09
GO:0001505	regulation of neurotransmitter levels	2.20232765566701e-08
GO:0046903	secretion	2.41874798015187e-08
GO:0019693	ribose phosphate metabolic process	2.59075933147293e-08
GO:0044708	single-organism behavior	3.75288426505447e-08
GO:0007268	chemical synaptic transmission	6.48812216855067e-08
GO:0098916	anterograde trans-synaptic signaling	6.48812216855067e-08
GO:0099536	synaptic signaling	6.48812216855067e-08
GO:0099537	trans-synaptic signaling	6.48812216855067e-08
GO:0006091	generation of precursor metabolites and energy	6.57931751816687e-08
GO:0032940	secretion by cell	7.81024422265308e-08
GO:0019637	organophosphate metabolic process	9.39659587790826e-08
GO:0019752	carboxylic acid metabolic process	9.79395777325743e-08
GO:0007269	neurotransmitter secretion	9.88634597159486e-08
GO:0099531	presynaptic process involved in chemical synaptic transmission	9.88634597159486e-08
GO:0099643	signal release from synapse	9.88634597159486e-08
GO:0006163	purine nucleotide metabolic process	1.00765997749304e-07
GO:0009117	nucleotide metabolic process	1.0430317824341e-07

d

Enriched GO BP terms for DE genes in *tud* mutant ovaries

GO:BP ID	Description	P-value
GO:0007304	chorion-containing eggshell formation	2.96530730212885e-05
GO:0030431	sleep	0.0282782633273288
GO:0097305	response to alcohol	0.029224173135073
GO:0035075	response to ecdysone	0.0302569154186064
GO:0036314	response to sterol	0.0302569154186064
GO:1901654	response to ketone	0.0302569154186064
GO:0014070	response to organic cyclic compound	0.0305498253481701
GO:0006635	fatty acid beta-oxidation	0.0339574133945106
GO:0071478	cellular response to radiation	0.0347159239260851
GO:0044712	single-organism catabolic process	0.0405250389252069
GO:0030258	lipid modification	0.0470151248572474

Supplementary Fig. 5: Functional classification of genes which are differentially expressed in *tudor* mutant brains and ovaries. a-d Most significantly enriched gene ontology (GO) biological process (BP) terms for differentially expressed (DE) genes in

tud mutant brains (**a**, **c**) and ovaries (**b**, **d**) are shown. In **a** and **b** the enriched GO terms names are listed on the Y-axis and corresponding -$\log_{10}$P values are plotted on the X-axis. In **c** and **d**, the enriched GO BP IDs are shown with the corresponding exact P-values.

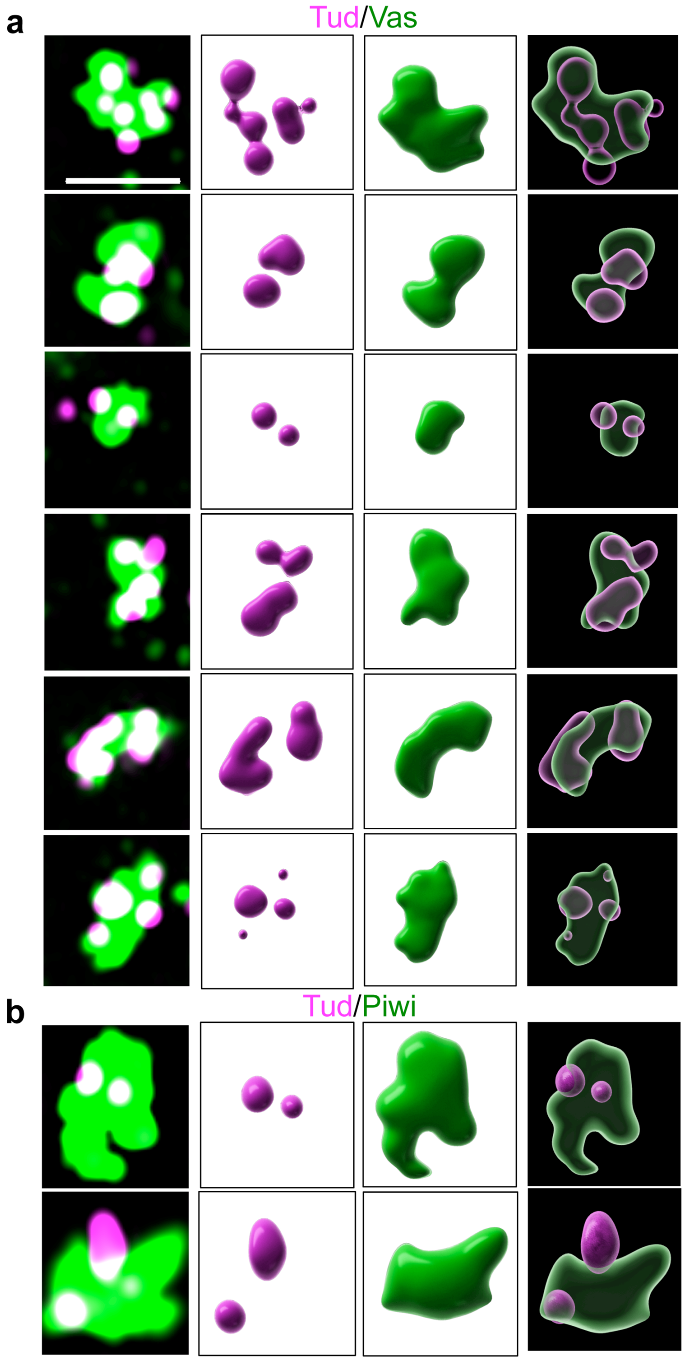

Supplementary Fig. 6: A gallery of Tud-, Vasa- and Piwi-containing glial granules. Overlays of super-resolution microscopy optical sections for different glial granules labeled with anti-FLAG (Tudor, magenta) and anti-Vas (**a**) or anti-Piwi (**b**) (green) are

shown in the left panels. Corresponding 3D reconstructions of Tud (magenta), Vas (**a**) or Piwi (**b**) (green) granule clusters (middle panels) and a composite granule (right panels) are shown. Scale bar shown in the first optical section (top left) is the same for all other granules' sections and is 2 μm.

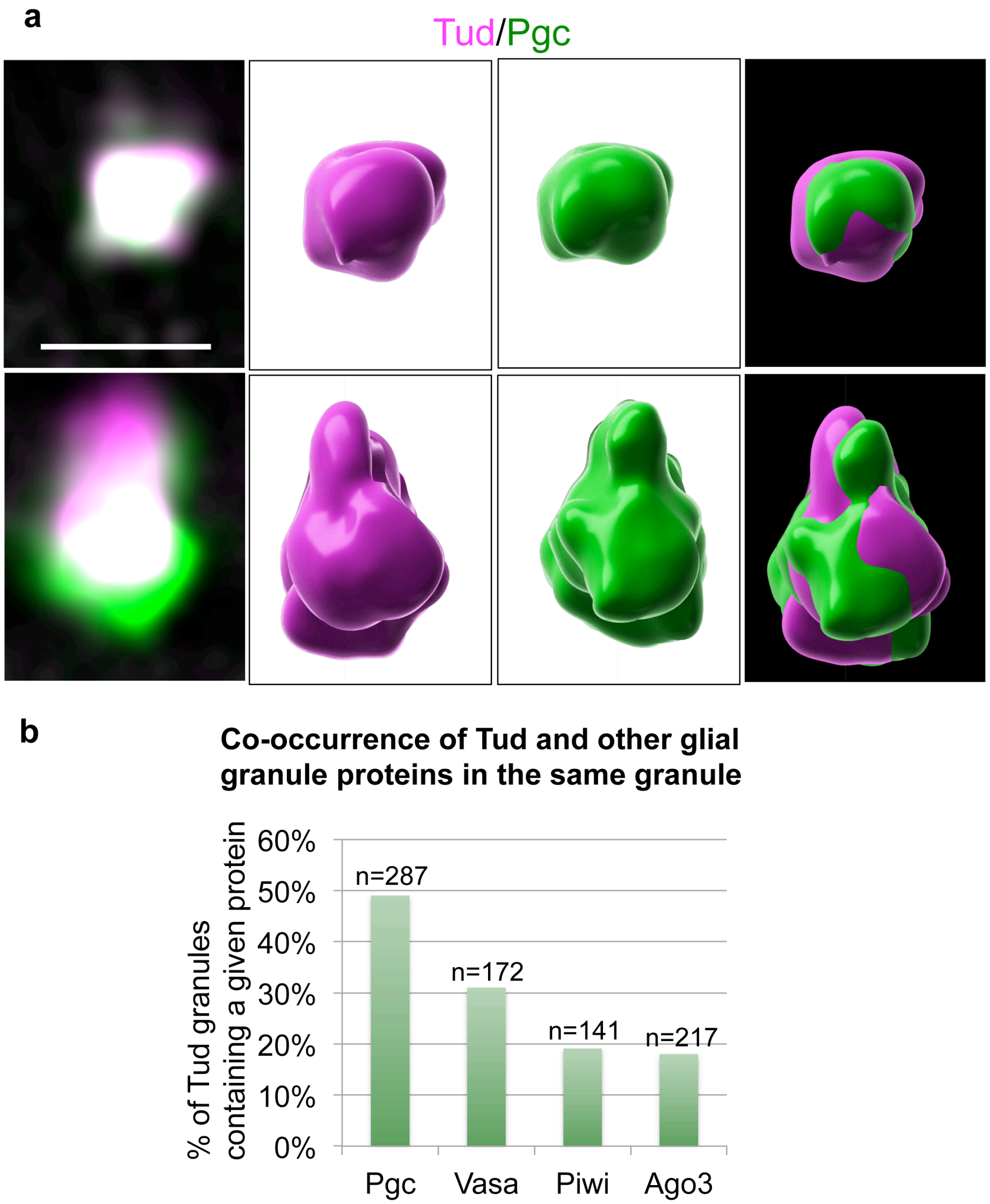

Supplementary Fig. 7: Tudor/Polar granule component-containing glial granules and quantification of co-occurrence of granule proteins in the same granule. a Overlays of super-resolution optical sections for two different glial granules labeled with anti-FLAG (Tudor, magenta) and anti-Pgc (green) are shown in the left panels. Corresponding 3D reconstructions of Tud (magenta) and Pgc (green) granule clusters (middle panels) and a composite granule (right panels) are shown. Scale bar is 2 μm. **b** Quantification of co-occurrence of Tud with Pgc, Vasa, Piwi and Ago3 in the same glial granules. Y-axis is the percentage of Tud granules that also contain a given protein

indicated on the X-axis (49%, 31%, 19% and 18% for Tud granules containing Pgc, Vasa, Piwi and Ago3 respectively). The number of granules used for the quantification is indicated for each protein.

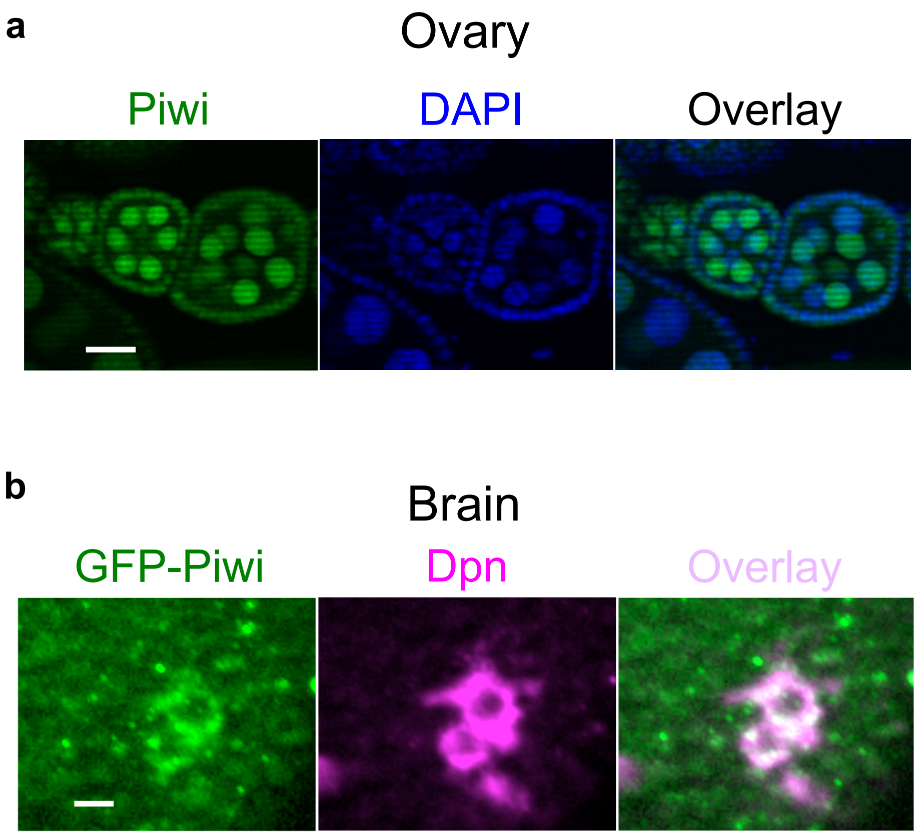

Supplementary Fig. 8: Piwi antibody used in the study shows the characteristic nuclear distribution in the egg chambers in the ovary and the genomic transgene GFP-Piwi labels the Dpn-positive cells in the adult brain. **a** Anti-Piwi antibody (green channel) shows characteristic labeling of nurse and follicle cell nuclei (DAPI, blue channel) in the developing egg chambers in the ovary. **b** Genomic transgene GFP-Piwi (green) labels Dpn-positive (magenta) cells in the adult brain similarly to anti-Piwi antibody (Figs. 4 and 5). Scale bars in **a** and **b** are 20 and 5 μm respectively.

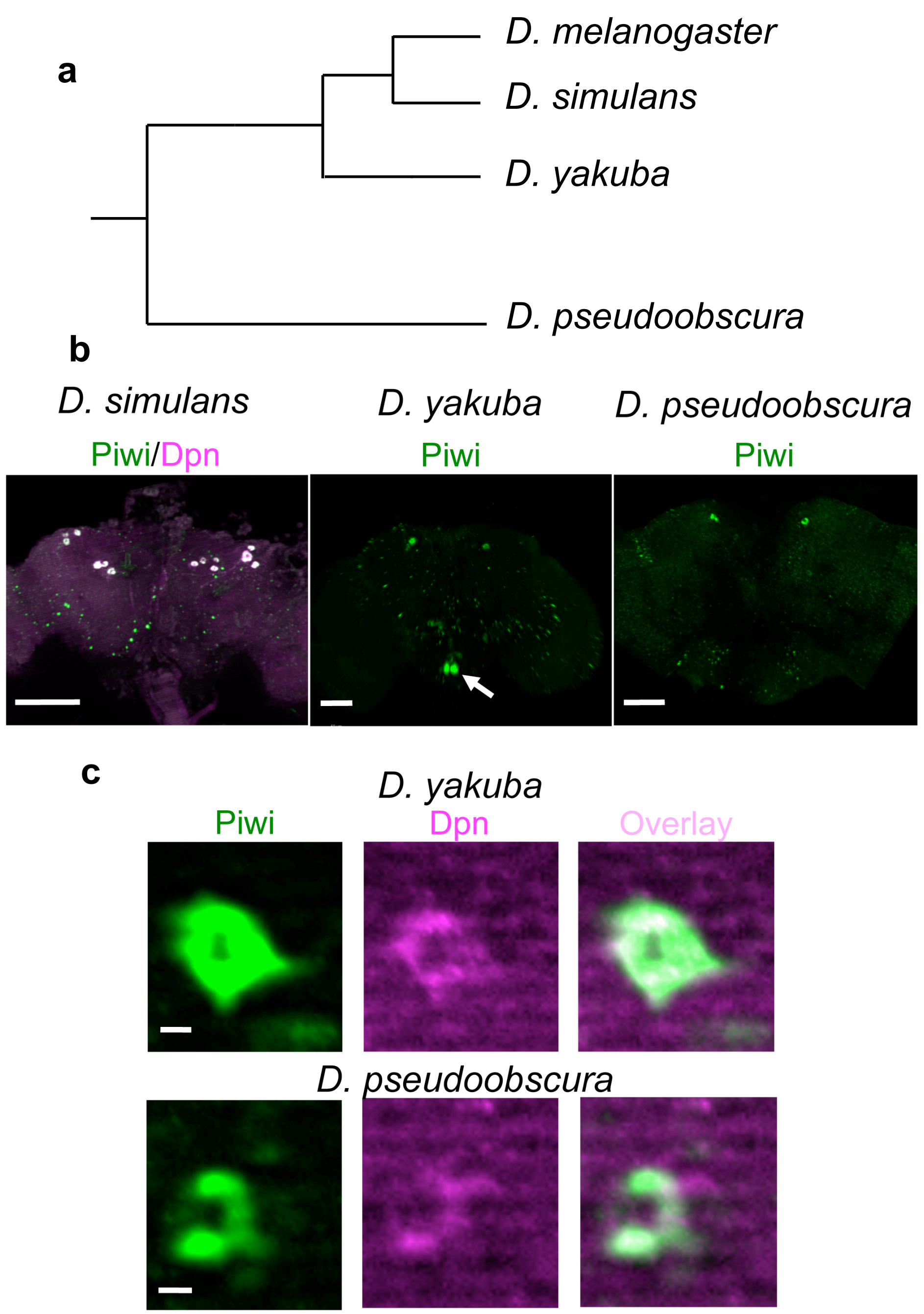

Supplementary Fig. 9: Piwi/Dpn-positive cells in other *Drosophila* species suggest their evolutionary conservation. a Phylogenetic tree of different *Drosophila* species used in this study. Divergence time from the common ancestor of these species is estimated at 25-55 million years. **b** Brains from *D. simulans* (left), *D. yakuba* (middle) and *D. pseudoobscura* (right) co-stained with anti-Piwi (green) and anti-Dpn (magenta) antibodies. In addition to Piwi-positive cells at the dorsal side, *D. yakuba*'s brains show ventrally located cells (arrow). Anti-Dpn antibody against *D. melanogaster* protein showed less reactivity toward *D. yakuba* and *D. pseudoobscura* Dpn, however, Dpn in the Piwi-positive cells was also detected in these species (**c**). Scale bars in **b** and **c** are 50 and 3 μm respectively.
