# Supplementary material for: Glial granules contain germline proteins in the Drosophila brain, which regulate brain transcriptome
Source: Commun Biol. 2020 Nov 20;3:699. doi: 10.1038/s42003-020-01432-z (PMC7679405; doi:10.1038/s42003-020-01432-z)
Supplement: Supplementary file 3 — Description of Additional Supplementary Files [file 42003_2020_1432_MOESM3_ESM.pdf]

Description of Additional Supplementary Files

File Name: Supplementary Data 1
Description: **Differentially expressed (DE) piRNAs in *tud* mutant brain**. All upregulated and top 100 downregulated piRNAs including their names, sequences and P-values are provided.

File Name: Supplementary Data 2
Description: **DE piRNAs in *tud* mutant ovary.** Top 100 up- and downregulated piRNAs including their names, sequences and P-values are provided.

File Name: Supplementary Data 3
Description: **Source data for DE transposable elements in *tud* mutant brain and ovary, and DE piRNAs that map to DE genes in *tud* mutant brain**. a, b log2 values of changes in the levels of DE transposable elements in tud mutants versus wild-type (log2FoldChange) with associated statistics parameters are shown for brain (a) and ovary (b). c log2 values of changes (tud mutant brains versus wild-type brains) in the levels of DE genes and DE piRNAs that map to these DE genes. Sense and antisense piRNAs are indicated as + and – respectively.

File Name: Supplementary Movie 1
Description: **Super-resolution imaging of glial granules containing Tudor and Ago3**. Z-stack volume of super-resolution optical sections show Tud-(red channel) and Ago3-(green channel) containing granules. This movie corresponds to Fig. 3f (note Ago3-specific labeling of the centrally located neuronal body indicated with arrow in Fig. 3f).

File Name: Supplementary Movie 2
Description: **Super-resolution imaging of a single glial granule containing Tudor and Polar granule component.** This movie shows progression through consecutive optical sections showing Tud (red channel) and Pgc (green channel) within a single glial granule.

File Name: Supplementary Movie 3
Description: **Location of Ago3-expressing neuronal bodies in the brain cortex**. Progression through optical sections of the adult brain showing Ago3-expressing clusters of neuronal bodies in the brain cortex (green channel). Representative Ago3-expressing cells are indicated with arrows.

File Name: Supplementary Movie 4
Description: **Detailed imaging of Ago3 expression in a subset of neuronal bodies in the cortex glia.** Progression through a super-resolution Zstack volume shows a subset of neuronal bodies expressing Ago3 (red channel) in cortex glia (green channel). Neuronal bodies' nuclei are labeled with DAPI (blue channel).

File Name: Supplementary Movie 5
Description: **3D reconstruction of Piwi/Dpn cells and extensions in the adult brain.** Piwi/Dpn-expressing cells show long extensions which 2 converge into the brain midline. 3D reconstruction was carried out using a Z-stack of super-resolution optical sections and related to Fig. 5a.

File Name: Supplementary Movie 6
Description: **Extensions emanating from Piwi/Dpn cells converge into Piwi-expressing cells in the brain midline.** Progression through optical sections of the adult brain showing Piwi-expressing cells and their extensions. Long extensions are converging to a cluster of Piwi-positive cells in the brain midline (indicated with arrows).

File Name: Supplementary Movie 7
Description: **Piwi/Dpn cell extensions can turn and continue along the brain midline.** Tracking Piwi-labeled extensions (green channel), with serial optical sectioning of the adult brain. The extensions exhibit sharp turns in the brain midline (indicated with arrows).

File Name: Supplementary Movie 8
Description: **Piwi/Dpn cells and extensions in *Drosophila simulans.*** Progression through serial optical sections of the D. simulans brain show Piwi-labeled cells and their extensions (green channel, indicated with arrows).
