# Supplementary material for: Glial granules contain germline proteins in the Drosophila brain, which regulate brain transcriptome
Source: Commun Biol. 2020 Nov 20;3:699. doi: 10.1038/s42003-020-01432-z (PMC7679405; doi:10.1038/s42003-020-01432-z)
Supplement: Supplementary file 15 — Reporting summary [file 42003_2020_1432_MOESM15_ESM.pdf]

nature research

Corresponding author(s): Alexey L. Arkov

Last updated by author(s): Oct 15, 2020

# Reporting Summary

Nature Research wishes to improve the reproducibility of the work that we publish. This form provides structure for consistency and transparency in reporting. For further information on Nature Research policies, see Authors & Referees and the Editorial Policy Checklist .

## Statistics

For all statistical analyses, confirm that the following items are present in the figure legend, table legend, main text, or Methods section.

| n/a | Confirmed | |
|---|---|---|
| [ ] | [x] | The exact sample size (*n*) for each experimental group/condition, given as a discrete number and unit of measurement |
| [ ] | [x] | A statement on whether measurements were taken from distinct samples or whether the same sample was measured repeatedly |
| [x] | [ ] | The statistical test(s) used AND whether they are one- or two-sided<br>*Only common tests should be described solely by name; describe more complex techniques in the Methods section.* |
| [x] | [ ] | A description of all covariates tested |
| [ ] | [x] | A description of any assumptions or corrections, such as tests of normality and adjustment for multiple comparisons |
| [x] | [ ] | A full description of the statistical parameters including central tendency (e.g. means) or other basic estimates (e.g. regression coefficient) AND variation (e.g. standard deviation) or associated estimates of uncertainty (e.g. confidence intervals) |
| [ ] | [x] | For null hypothesis testing, the test statistic (e.g. *F*, *t*, *r*) with confidence intervals, effect sizes, degrees of freedom and *P* value noted<br>*Give P values as exact values whenever suitable.* |
| [x] | [ ] | For Bayesian analysis, information on the choice of priors and Markov chain Monte Carlo settings |
| [x] | [ ] | For hierarchical and complex designs, identification of the appropriate level for tests and full reporting of outcomes |
| [x] | [ ] | Estimates of effect sizes (e.g. Cohen's *d*, Pearson's *r*), indicating how they were calculated |

*Our web collection on statistics for biologists contains articles on many of the points above.*

## Software and code

Policy information about availability of computer code

| | |
|---|---|
| Data collection | For LSM 880 confocal and superresolution imaging with Airyscan, ZEN 2.3 version 14.0.18.201 (Zeiss) was used. |
| Data analysis | For imaging analysis and 3D reconstructions, Imaris (version 9.5, Oxford Instruments) was run on HP Z8 workstation. For differential gene expression, software used includes tophat2 (v2.0.3), cuffdiff (v2.2.1). Differential expression of piRNA used edgeR (v3.16.5) and PPmeter (v0.4). |

For manuscripts utilizing custom algorithms or software that are central to the research but not yet described in published literature, software must be made available to editors/reviewers. We strongly encourage code deposition in a community repository (e.g. GitHub). See the Nature Research guidelines for submitting code & software for further information.

## Data

Policy information about availability of data

All manuscripts must include a data availability statement. This statement should provide the following information, where applicable:

- Accession codes, unique identifiers, or web links for publicly available datasets
- A list of figures that have associated raw data
- A description of any restrictions on data availability

Small RNA sequencing (piRNA) and RNA-Seq data is available in GEO superseries GSE149750 https://www.ncbi.nlm.nih.gov/geo/query/acc.cgi?acc=GSE149750

# Field-specific reporting

Please select the one below that is the best fit for your research. If you are not sure, read the appropriate sections before making your selection.

# Life sciences study design

All studies must disclose on these points even when the disclosure is negative.

| | |
|---|---|
| Sample size | No statistical methodology was used to predetermine sample size. However, the number of samples for every experimental condition or treatment used in this study was estimated based on the methodology previously established in the field and is described in the Methods section and figure legends. |
| Data exclusions | No data were excluded in this manuscript. |
| Replication | Experimental data reported in this study were successfully reproduced. Specific description of the number of the replicates and samples used is provided in the Methods section and figure legends. |
| Randomization | Allocation of samples into experimental groups was not intentionally ordered or randomized. |
| Blinding | Investigators were not blinded to allocation since the same individuals both collected and analyzed the data. |

# Reporting for specific materials, systems and methods

We require information from authors about some types of materials, experimental systems and methods used in many studies. Here, indicate whether each material, system or method listed is relevant to your study. If you are not sure if a list item applies to your research, read the appropriate section before selecting a response.

## Materials & experimental systems

| n/a | Involved in the study |
|---|---|
| ☐ | ☒ Antibodies |
| ☒ | ☐ Eukaryotic cell lines |
| ☒ | ☐ Palaeontology |
| ☐ | ☒ Animals and other organisms |
| ☒ | ☐ Human research participants |
| ☒ | ☐ Clinical data |

## Methods

| n/a | Involved in the study |
|---|---|
| ☒ | ☐ ChIP-seq |
| ☒ | ☐ Flow cytometry |
| ☒ | ☐ MRI-based neuroimaging |

## Antibodies

| | |
|---|---|
| Antibodies used | Rabbit anti-GFP (Abcam, ab290, dilution 1:5000); mouse anti-FLAG (Sigma, F1804, dilution 1:5000); rabbit anti-FLAG (Novus Biologicals, NB600-345, dilution 1:4000); mouse anti-Repo (DSHB, 8D12 anti-Repo-s, was deposited to the DSHB by C. Goodman, dilution 1:200), guinea pig anti-Repo (B. Altenhein, University of Cologne, dilution 1:2500), mouse anti-Wrapper (DSHB, 10D3 anti-wrapper-s, was deposited to the DSHB by C. Goodman, dilution 1:400); rabbit anti-Vas (R. Lehmann, HHMI, dilution 1:2000); rabbit anti-Pgc (A. Nakamura, Kumamoto University, dilution 1:2000); rabbit anti-Ago3 (G. Hannon, University of Cambridge, dilution 1:1500); rabbit anti-Piwi (Sdix, distributed by Novus Biologicals, 40300002, dilution 1:1500); guinea pig anti-Dpn (J. Knoblich, IMBA, dilution 1:2400). |
| Validation | The commercial anti-GFP and anti-FLAG antibodies have been validated and published and full antibody profiles can be found in https://www.abcam.com/gfp-antibody-chip-grade-ab290.html, https://www.sigmaaldrich.com/catalog/product/sigma/f1804?lang=en&region=US&gclid=EAIaIQobChMI_v_JsJqd6QIVD9vACh1Kzgk2EAAYASAAEgIVA_D_BwE and https://www.novusbio.com/products/dykddddk-epitope-tag-antibody_nb600-345#reviews-publications. Also, the commercial anti-Repo antibody and anti-Wrapper antibody have been validated and published and full antibody profile is provided in https://dshb.biology.uiowa.edu/8D12-anti-Repo and https://dshb.biology.uiowa.edu/10D3-anti-wrapper. In addition, the commercial anti-Piwi antibody has been validated in the manuscript Supplementary Fig. 8. All other antibodies used in the manuscript have been validated and published by others (see corresponding references for each antibody in the Methods section). |

## Animals and other organisms

Policy information about studies involving animals; ARRIVE guidelines recommended for reporting animal research

| | |
|---|---|
| Laboratory animals | Drosophila melanogaster, simulans, yakuba and pseudoobscura. |
| Wild animals | The study did not involve wild animals. |
| Field-collected samples | The study did not involve field-collected samples. |
| Ethics oversight | No formal ethical approval was required. |

Note that full information on the approval of the study protocol must also be provided in the manuscript.
